# A prolonged chronological lifespan is an unexpected benefit of the [*PSI*^+^] prion in yeast

**DOI:** 10.1371/journal.pone.0184905

**Published:** 2017-09-14

**Authors:** Kai Wang, Ronald Melki, Mehdi Kabani

**Affiliations:** Paris-Saclay Institute of Neuroscience, Centre National de la Recherche Scientifique, Université Paris-Saclay, Gif-sur-Yvette, France; Deutsches Zentrum fur Neurodegenerative Erkrankungen, GERMANY

## Abstract

Self-replicating ‘proteinaceous infectious particles’ or prions are responsible for complex heritable traits in the yeast *Saccharomyces cerevisiae*. Our current understanding of the biology of yeast prions stems from studies mostly done in the context of actively dividing cells in optimal laboratory growth conditions. Evidence suggest that fungal prions exist in the wild where most cells are in a non-dividing quiescent state, because of imperfect growth conditions, scarcity of nutrients and competition. We know little about the faithful transmission of yeast prions in such conditions and their physiological consequences throughout the lifespan of yeast cells. We addressed this issue for the [*PSI*^+^] prion that results from the self-assembly of the translation release factor Sup35p into insoluble fibrillar aggregates. [*PSI*^+^] leads to increased nonsense suppression and confers phenotypic plasticity in response to environmental fluctuations. Here, we report that while [*PSI*^+^] had little to no effect on growth *per se*, it dramatically improved the survival of yeast cells in stationary phase. Remarkably, prolonged chronological lifespan persisted even after [*PSI*^+^] was cured from the cells, suggesting that prions may facilitate the acquisition of complex new traits. Such an important selective advantage may contribute to the evolutionary conservation of the prion-forming ability of Sup35p orthologues in distantly related yeast species.

## Introduction

The yeast *Saccharomyces cerevisiae* hosts many ‘infectious proteins’, or prions, with a striking enrichment in proteins involved in gene regulation at the transcriptional or translational levels [1]. The discovery and characterization of [*PSI*^+^] allowed tremendous progress on our current understanding of the molecular processes that determine the formation and maintenance of prions, and also of the possible biological properties conferred by infectious protein particles [2,3]. [*PSI*^+^] results from the assembly of the translation termination factor Sup35p into insoluble self-replicating fibrillar protein aggregates [2,3]. [*PSI*^+^] cells exhibit an increased level of translational readthrough due to reduced translation termination efficacy [4,5]. Furthermore, structurally distinct Sup35p assemblies lead to different [*PSI*^+^] strains which are referred to as ‘weak’ or ‘strong’ with respect to the nonsense suppression phenotypes they confer [3]. As a consequence of both altered translation termination and the cellular response to prion aggregates, [*PSI*^+^] and prion-free [*psi*^-^] cells most likely have different proteomes [6]. [*PSI*^+^] was proposed to function as a transient evolutionary capacitator allowing the acquisition and genetic fixation of new traits, particularly in stressful environmental conditions [7–10]. However, whether [*PSI*^+^] strains are beneficial or mostly detrimental for yeast is still a debated issue [3,11–17].

Most of the knowledge acquired on yeast prions in general and [*PSI*^+^] in particular, results from studies on actively dividing yeast cells grown in optimal laboratory conditions. In nature, cells are frequently found in a non-proliferating quiescent state, for example when nutrient sources are scarce or when environmental conditions (e.g. temperature) are not favorable to growth [18]. The ability of yeasts to enter into quiescence, a physiological state characterized by specific cell structures such as actin bodies or proteasome storage granules as well as an increased resistance to stress and external aggressions, is undoubtedly a key factor for chronological lifespan extension in this organism [19,20].

Here, we show in different genetic backgrounds that [*PSI*^+^] cells have a much longer chronological lifespan compared to [*psi*^-^] cells. Strikingly, curing cells from [*PSI*^+^] did not alleviate this beneficial effect suggesting prions can elicit the acquisition of new traits. The ability of cells to survive much longer in starvation conditions certainly confers a selective advantage that could explain the presence of [*PSI*^+^] among wild yeasts and the evolutionary conservation of the prion-forming ability of Sup35p in distantly related yeast species.

## Results

**Fig 1:**
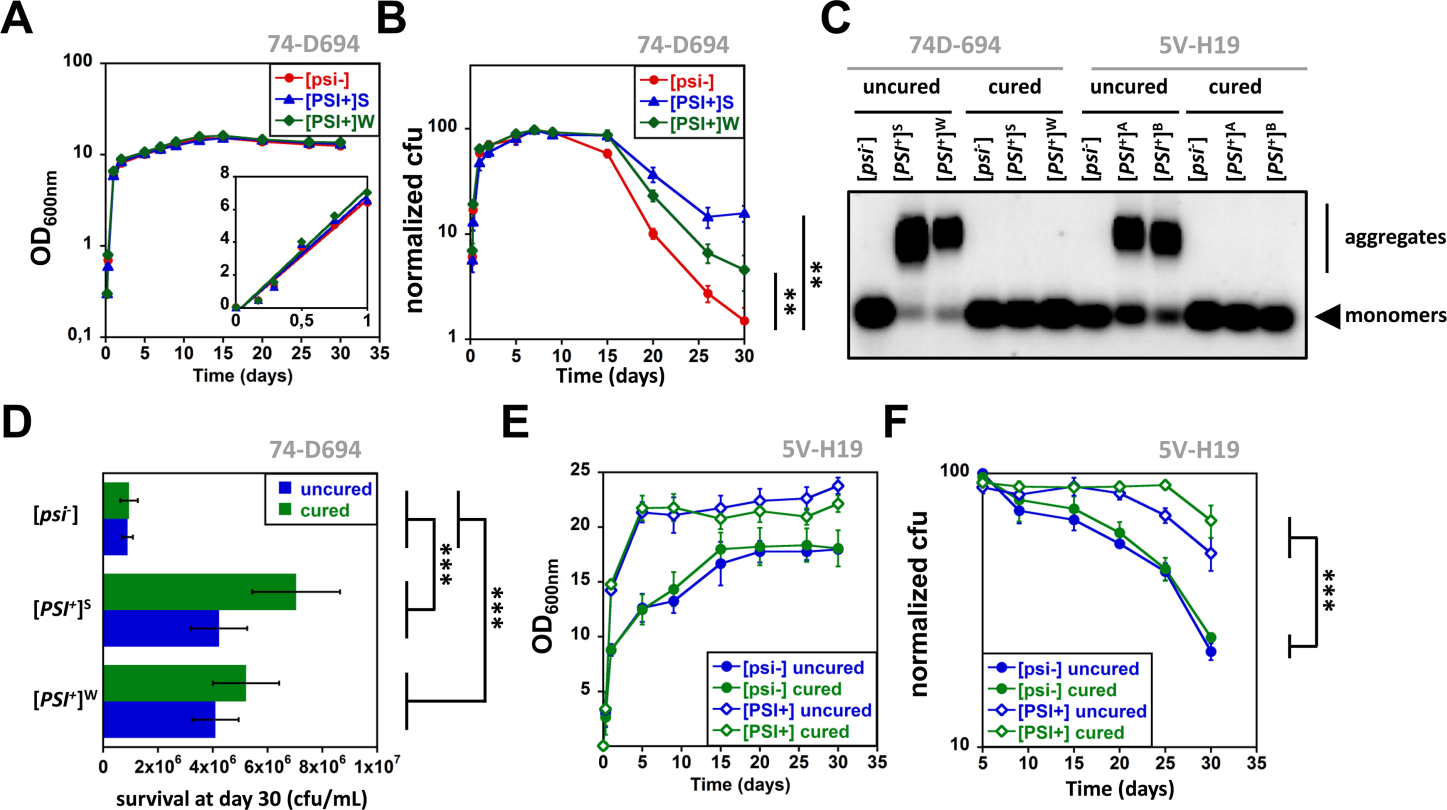
[*PSI*^+^] confers a prolonged chronological lifespan in both the 74-D694 and 5V-H19 genetic backgrounds. **(A)** Exponentially growing [*psi*^-^], [*PSI*^+^]^S^ or [*PSI*^+^]^W^ 74-D694 cells were inoculated in fresh YPDA medium and allowed to grow for up to 30 days at 30°C under agitation. Aliquots were periodically removed to measure the optical density at 600 nm (data are the mean ±SE of five independent cultures; error bars are within the variables symbols). The inset shows a magnified portion of the growth curve. **(B)** Same cultures as in (A). The number of cfu was determined by serial dilutions and plating. Growth and survival curves were normalized with respect to the maximal number of cfu set to 100% (non-normalized data can be found in S1A Fig). Data points are the mean ±SE of five independent cultures. Statistical comparison of the growth curves was performed using a permutation test (see Materials and Methods; ** indicate p-values < 0.01). **(C)** Cell lysates from the indicated 74-D694 or 5V-H19 strains were analyzed by SDD-AGE followed by immunoblotting using anti-Sup35p antibodies. The position of Sup35p monomers (fast migrating species) and aggregates (slow migrating species) is indicated. **(D)** Exponentially growing [*psi*^-^], [*PSI*^+^]^S^ or [*PSI*^+^]^W^ 74-D694 cells, that were previously cured with guanidine hydrochloride or not, as indicated, were inoculated in YPDA medium and incubated at 30°C under agitation for 30 days. The number of cfu was then determined by serial dilutions and plating. Data represent the mean ±SE of three independent cultures (*** indicate p-values < 0.001, unpaired two-tailed Student’s t-test).**(E)** Exponentially growing [*psi*^-^] or [*PSI*^+^] 5V-H19 cells were inoculated in fresh YPDA medium and allowed to grow for up to 30 days at 30°C under agitation. Aliquots were periodically removed to measure the optical density at 600 nm (data are the mean ±SE of four to six independent cultures). **(F)** Same cultures as in (E). The number of cfu was determined by serial dilutions and plating. Survival curves were normalized with respect to the maximal number of cfu set to 100% (non-normalized data can be found in S1B Fig). Data points are the mean ±SE of four to six independent cultures. Statistical comparison of the growth curves was performed using a permutation test (see Materials and Methods; *** indicate p-values < 0.001).

We first rigorously compared the growth rates of [*psi*^-^] and [*PSI*^+^] derivatives of the 74-D694 yeast strain in rich YPDA medium. Cell growth was assessed by measuring the optical density at 600nm (OD_600nm)_ and the number of colony-forming units (cfu) (see Materials and Methods section). To enable a proper comparison between [*psi*^-^] and [*PSI*^+^] strains, we made sure that all cultures were started with exponentially growing cells, preadapted to the culture conditions (see Materials and Methods section). As a result, no lag phase preceding growth was observed upon diluting cells in fresh medium (Fig 1A). The growth rates of [*psi*^-^] and [*PSI*^+^] strains were indistinguishable (Fig 1A, *inset*) and yielded the same maximal numbers of cfu after 7 days of growth (Fig 1B and S1A Fig). Cfu numbers correlated linearly with optical density measurements only during early growth (until OD_600nm_~12) (S2 Fig). Thus, Sup35p prion aggregates, whether propagating strong or weak [*PSI*^+^] variants, are neither detrimental nor beneficial for growth in the 74-D694 yeast strain, as previously reported [9]. [*psi*^-^] and [*PSI*^+^] stationary phase cells remained perfectly viable for 7 more days of incubation (Fig 1B). The [*PSI*^+^] prion was stably maintained in both strong and weak [*PSI*^+^] strains, while no spontaneous [*PSI*^+^] clones appeared in the [*psi*^-^] population (S3 Fig).

A gradual decay in cfu numbers, which could be due to cell death, senescence and/or an inability of aged cells to exit the quiescent state, was observed for all cultures after 15 days of growth (Fig 1B). Remarkably, the loss of viability was much greater for [*psi*^-^] cells than for [*PSI*^+^]^S^ or [*PSI*^+^]^W^ cells (Fig 1B and S1A Fig). From day 7 to day 30, cfu numbers dropped from 100% (~5x10^7^ cfu/mL) to ~15% (~7x10^6^ cfu/mL) for [*PSI*^+^]^S^ cultures, to ~5% (~2x10^6^ cfu/mL) for [*PSI*^+^]^W^ cultures and to ~1% (~7x10^5^ cfu/mL) for [*psi*^-^] cultures (Fig 1B and S1A Fig). Thus, the [*PSI*^+^] prion appears to have a positive effect on yeast chronological lifespan. Microscopic analysis of the cultures at the different stages of growth did not reveal significant qualitative or quantitative morphological differences between [*psi*^-^] and [*PSI*^+^] cells (data not shown). Treating cells with guanidine hydrochloride effectively cured them from the [*PSI*^+^] prion as witnessed by *i-* the pink or white to red change of the clones color on adenine-limiting medium (S4A Fig) and *ii-* by the conversion of the slow migrating aggregated Sup35p species to fast migrating soluble Sup35p species (Fig 1C). This treatment affected neither the growth properties nor the chronological lifespan of [*psi*^-^] cells (Fig 1D and data not shown). Importantly however, the chronological lifespan of uncured and cured [*PSI*^+^] cells was similar and significantly higher than that of the original [*psi*^-^] cells (Fig 1D). Cured cells retained their [*psi*^-^] status throughout the 30 days of culture, as shown by their red colony color after plating on adenine-limiting medium (S5A Fig) and by the absence of Sup35p aggregates visible in SDD-AGE assays (S5B Fig). This indicates that the beneficial effect of [*PSI*^+^] on yeast chronological lifespan can be maintained in the cells even when [*PSI*^+^] is subsequently lost [7,10].

The biological consequences and phenotypic manifestations of genetic and epigenetic polymorphisms (e.g. mutations, prions) can vary greatly depending on the genetic background of the yeast strains in which they are studied. To address whether the beneficial effect conferred by [*PSI*^+^] on chronological lifespan is specific to the 74-D694 strain context or not, we compared the growth properties and chronological lifespan of [*psi*^-^] and freshly obtained [*PSI*^+^] strains in the 5V-H19 background (see Materials and Methods section). [*PSI*^+^] 5V-H19 cells grew slightly faster and generated a greater number of cfu (~1x10^8^ cfu/mL vs ~8x10^7^ cfu/mL) than their [*psi*^-^] cells counterparts, in agreement with previous data [9] (Fig 1E and S1B Fig). Survival rates of 5V-H19 [*PSI*^+^] cells (~55%; ~6x10^7^ cfu/mL) in stationary phase conditions were twice as high as those of 5V-H19 [*psi*^-^] cells (~25%; ~2x10^7^ cfu/mL) (Fig 1F and S1B Fig). As for 74-D694 cells, treating 5V-H19 cells with guanidine hydrochloride cured them from the [*PSI*^+^] prion (Fig 1C and S4B Fig). Once again, the survival rates of cured 5V-H19 [*PSI*^+^] cells (~67%; 8.5x10^7^ cfu/mL) remained higher than those of both the original and guanidine hydrochloride cured 5V-H19 [*psi*^-^] strains, the latter behaving similarly in these assays (~25%; ~2x10^7^ cfu/mL) (Fig 1F and S1B Fig). Furthermore, cured 5V-H19 [*PSI*^+^] cells retained higher growth rates than uncured and guanidine hydrochloride cured 5V-H19 [*psi*^-^] cells (Fig 1E). Thus, in a manner similar to what we report for 74-D694 cells, [*PSI*^+^] confers a selective advantage to 5V-H19 cells by improving both their growth rate and chronological lifespan. This selective advantage is subsequently maintained in the cells and becomes [*PSI*^+^]-independent. To further strengthen our observations, chronological lifespan assays were repeated in uncured and cured [*psi*^-^] and [*PSI*^+^] G402-A1 cells [21] leading to the same conclusions (S6 Fig).

## Discussion

Most weak or strong [*PSI*^+^] variants obtained and studied in laboratory conditions are well tolerated by yeast cells and stably maintained during cell divisions with no or little growth impairment (e.g. Fig 1A and 1E) [9]. [*PSI*^+^] formation is associated with reduced translational fidelity which leads to genome-wide nonsense suppression events [6]. [*PSI*^+^]-induced changes in gene expression facilitate the phenotypic expression of cryptic genetic polymorphisms, leading to a variety of acquired traits which are often beneficial under challenging growth conditions [7–10]. Here we find that one of such [*PSI*^+^]-induced traits is the ability to survive longer when nutrients are exhausted (Fig 1, S1 Fig and S6 Fig). Remarkably, [*PSI*^+^]-mediated long-term survival was robustly observed using different prion variants in three very different genetic backgrounds (Fig 1, S1 Fig and S6 Fig). It is important to highlight that we used [*PSI*^+^] strains of different origins as well as [*PSI*^+^] cells that were freshly obtained by transforming [*psi*^-^] cells with infectious prions (Fig 1, S1 Fig and S6 Fig). In all cases, the effects we observe on survival rates are consistent and only differ in amplitude depending on the genetic background of the strains and the prion variants they carry (Fig 1, S1 Fig and S6 Fig). Therefore, it is unlikely that they are due to genetic mutations present in our strains, unless such mutations arise or manifest as a consequence of [*PSI*^+^]-induced changes in gene expression [6]. Furthermore, the chronological lifespan of 74-D694 [*psi*^-^] cells subjected to a spheroplast transformation procedure but that were not converted to [*PSI*^+^] was similar (5x10^5^-1x10^6^ cfu/mL at day 30) to that of the naïve 74-D694 [*psi*^-^] cells we used throughout this study (S1A Fig). This indicates that the longer lifespan of [*PSI*^+^] cells is not an indirect consequence of the prion conversion process. Nutrient scarcity is probably one of the most frequent stressful conditions faced by yeasts in nature. A prolonged chronological lifespan allowing cells to survive longer under such conditions would be a major selective advantage for yeasts in the wild, which could explain the extraordinary conservation of the prion-forming ability of Sup35p over one billion years of fungal evolution [22]. In agreement with our own findings, Speldewinde and Grant recently reported that [*PSI*^+^] improves chronological lifespan and proposed this could be due to an increased autophagic flux [23].

The permanent genetic fixation of [*PSI*^+^] dependent traits was previously described and thought to occur mainly by the re-assortment of genetic variation during meiosis [7,10]. Here we made the striking observation that haploid 74-D694, 5V-H19 and G402-A1 cells cured from [*PSI*^+^] with guanidine hydrochloride retained a prolonged chronological lifespan in stationary phase conditions (Fig 1, S1 Fig and S6 Fig). Importantly, curing had no effect on the survival rates of naïve [*psi*^-^] cells, indicating that neither the guanidine hydrochloride treatment nor other prions that may have been initially present in the cells are responsible for these observations (Fig 1, S1 Fig and S6 Fig). In contrast with our findings, Speldewinde and Grant reported that cured cells had a shorter lifespan compared to [*PSI*^+^] cells [23]. It is important to note that these authors used five rounds of growth on guanidine hydrochloride-containing medium to cure cells [23], a harsh and selective treatment that could have unknown side-effects, as opposed to our gentler curing conditions sufficient to eliminate [*PSI*^+^] (Fig 1C, S4 Fig and S5 Fig), through two passages on guanidine hydrochloride-containing medium (see Materials and Methods section). It is worth noting that the prion curing method we used neither affected the growth properties nor the chronological lifespan of [*psi*^-^] cells (Fig 1, S1 Fig and S6 Fig).

If the selective advantage conferred by [*PSI*^+^] for long-term survival was a rare stochastic event arising in only a few cells within the population, it should have passed undetected in our experiments. Thus, while we believe that [*PSI*^+^] could act on aging by promoting an increase in autophagic flux as suggested [23], it might also trigger a genetic and/or metabolic reprogramming that remains to be elucidated. For instance, [*PSI*^+^] could act on Tor/Sch9 and/or RAS/adenylate cyclase/PKA, the two major signaling pathways that regulate lifespan in yeast [20,24]. In the absence of meiotic re-assortment of polymorphisms and alleles in haploid cells, we propose that this [*PSI*^+^]-dependent mechanism could be of epigenetic essence, such as the induction of a new pro-aging prion refractory to guanidine hydrochloride curing [25].

It has become clear that protein-based inheritance plays a significant role in the acquisition of new -often beneficial- phenotypic states in yeast, the importance of which may be still largely underestimated because of yeast domestication in standardized laboratory conditions [7,10,25,26].

## Materials and methods

### Yeast strains, growth media and monitoring of prion phenotypes

The *S*. *cerevisiae* strains used in this study were derived from 74-D694 [MATa *ade1-14* (UGA) *trp1-289 leu2-3*,*112 his3*Δ*-200 ura3-52*], 5V-H19 [MATα *SUQ5 ade2-1*(UAA) *can1-100*, *leu2-3*,*112*, *ura3-52*] or *G402-A1* [MATa, *kar1–1*, *SUQ5*, *ade2-1*, *his3Δ202*, *leu2Δ1*, *lys2*, *trp1Δ63*, *ura3-52*, *ssa1*::*KanMX*, *ssa2*::*-HIS3*, *ssa3*::*TRP1*, *ssa4*::*ura3-1f*/pRDW10] [21]. When indicated, these strains carried strong ([*PSI*^+^]^S^) or weak ([*PSI*^+^]^W^) [*PSI*^+^] prion variants, or no prion ([*psi*^-^]). 5V-H19 [*PSI*^+^] strains were obtained by protein infection as described before [27,28]. Yeast cells were grown in YPDA medium (1% yeast extract, 2% peptones, 2% glucose, 0.002% adenine). Solid media contained 2% bacto-agar. Prion phenotypes were monitored on ¼-YPD medium (0.25% yeast extract, 2% peptones, 2% glucose) via a standard color-based phenotype assay, as described before [27–29]. To cure them from prions, cells were passaged twice on YPD plates containing 3 mM guanidine hydrochloride and then once on ¼-YPD plates, as described before [27,28]. Cured cells were tested for growth on non-fermentable carbon sources to exclude *petite* mutants that may arise from the guanidine hydrochloride treatment [30].

### Chronological lifespan assays

The indicated strains were grown overnight at 30°C under agitation in YPDA medium. The following day, these cultures were diluted into fresh medium and cells were allowed to divide 3–4 times at 30°C under agitation before being diluted in fresh medium again. This procedure was repeated again and ensured that all analyzed cultures were started with cells in the exponential phase of growth. Cultures were then incubated at 30°C under agitation for up to 30 days and 100 μL to 1 mL aliquots were removed at the indicated time points. Tenfold serial dilutions of these aliquots were then plated in duplicate on ¼-YPD plates to count the colony-forming units (cfu) and assess the prion phenotypes of the cells. The OD_600nm_ was also measured at each time point. When indicated, survival curves were normalized according to the maximal number of cfu reached by each culture which was set to 100%.

The statistical permutation tests used to perform the pairwise comparison of groups of sFurvival curves from different strains were performed on both non-normalized and normalized data using the *Compare Groups of Growth Curves* web interface (http://bioinf.wehi.edu.au/software/compareCurves), which uses the *growthcurve* function from the *statmod* software package for *R*, and according to the author’s instructions [31,32].

### Preparation of cell extracts and semi-denaturant detergent agarose gel electrophoresis (SDD-AGE)

Yeast cells (~20 OD_600nm_ units) were harvested by centrifugation at 4000 g for 2 min and resuspended in 500 μl of lysis buffer (100 mM Tris-Cl pH 7.5, 50 mM KCl, 10 mM β-mercaptoethanol, 1 mM PMSF and protease inhibitor cocktail, Roche Diagnostics). Glass beads were added to half the cell suspension volume and cells were broken by six cycles of vortexing for 30 s with 1 min incubation on ice between each vortexing. Debris and unbroken cells were removed by centrifugation for 2 min at 4000 x g and at 4°C. SDD-AGE analysis was performed as described previously [27,28].

## Supporting information

S1 Fig [*PSI*^+^] confers a prolonged chronological lifespan in the 74-D694 and 5V-H19 backgrounds. **(A)** Same as Fig 1B. Data points are the mean cfu numbers ±SE of five independent cultures. Statistical comparison of the growth curves was performed using a permutation test (see Materials and Methods; ** indicate p-values < 0.01). **(B)** Same as Fig 1F. Data points are the mean cfu numbers ±SE of four to six independent cultures. Statistical comparison of the growth curves was performed using a permutation test (see Materials and Methods; ** indicate p-values < 0.01).
(TIF)

S2 Fig OD_600nm_ measurements correlation with cfu numbers in [*psi*^-^] and [*PSI*^+^] 74-D694 cells. A linear correlation between optical density measurements and cfu numbers (data points are from Fig 1A and 1B) was observed at OD_600nm_<12.
(TIF)

S3 Fig The [*PSI*^+^] prion is stably maintained during stationary phase. [*psi*^-^], [*PSI*^+^]^S^ or [*PSI*^+^]^W^ 74-D694 cells incubated at 30°C under agitation for up to 30 days were periodically plated on ¼-YPD plates to assess their prion phenotypes. As expected, no spontaneous formation of [*PSI*^+^] colonies occurred in the [*psi*^-^] cultures. The [*PSI*^+^]^S^ variant was stably maintained in all cells while the [*PSI*^+^]^W^ variant was lost in less than 5% of the cells.
(TIF)

S4 Fig Guanidine hydrochloride-treated cells are cured from the [*PSI*^+^] prion. Representative **(A)** 74-D694 (*W*, weak [*PSI*^+^]; *S*, strong [*PSI*^+^]) or **(B)** 5V-H19 ([*PSI*^+^] clone *A*; lower panel) [*psi*^-^] and [*PSI*^+^] clones, either left untreated or treated with guanidine hydrochloride (see Materials and Methods), were streaked on ¼-YPD plates to assess their prion phenotypes.
(TIF)

S5 Fig Guanidine hydrochloride-cured cells retain their [*psi*^-^] status after 30 days of culture. Exponentially growing uncured or guanidine hydrochloride-cured [*PSI*^+^]^W^ or [*PSI*^+^]^S^ 74-D694 cells were inoculated into fresh YPDA medium and incubated at 30°C under agitation for 30 days. **(A)** Aliquots of the cultures were plated on ¼-YPD plates to assess their prion phenotypes. **(B)** Cell lysates prepared from each culture were analyzed by SDD-AGE followed by immunoblotting using anti-Sup35p antibodies. The position of Sup35p monomers (fast migrating species) and aggregates (slow migrating species) is indicated.
(TIF)

S6 Fig [*PSI*^+^] confers a prolonged chronological lifespan in the G402-A1 strain. Exponentially growing [*psi*^-^] and [*PSI*^+^] G402-A1 cells, that were previously cured with guanidine hydrochloride or not, as indicated, were inoculated in fresh YPDA medium and incubated at 30°C under agitation for 30 days. The number of cfu was then determined by serial dilutions and plating. Data represent the mean ±SE of four independent cultures (*** indicate p-values <0.001, unpaired two-tailed Student’s t-test).
(TIF)
