# Supplementary figures and images for: A prolonged chronological lifespan is an unexpected benefit of the [PSI+] prion in yeast
Source: PLoS One. 2017 Sep 14;12(9):e0184905. doi: 10.1371/journal.pone.0184905 (PMC5599042; doi:10.1371/journal.pone.0184905)

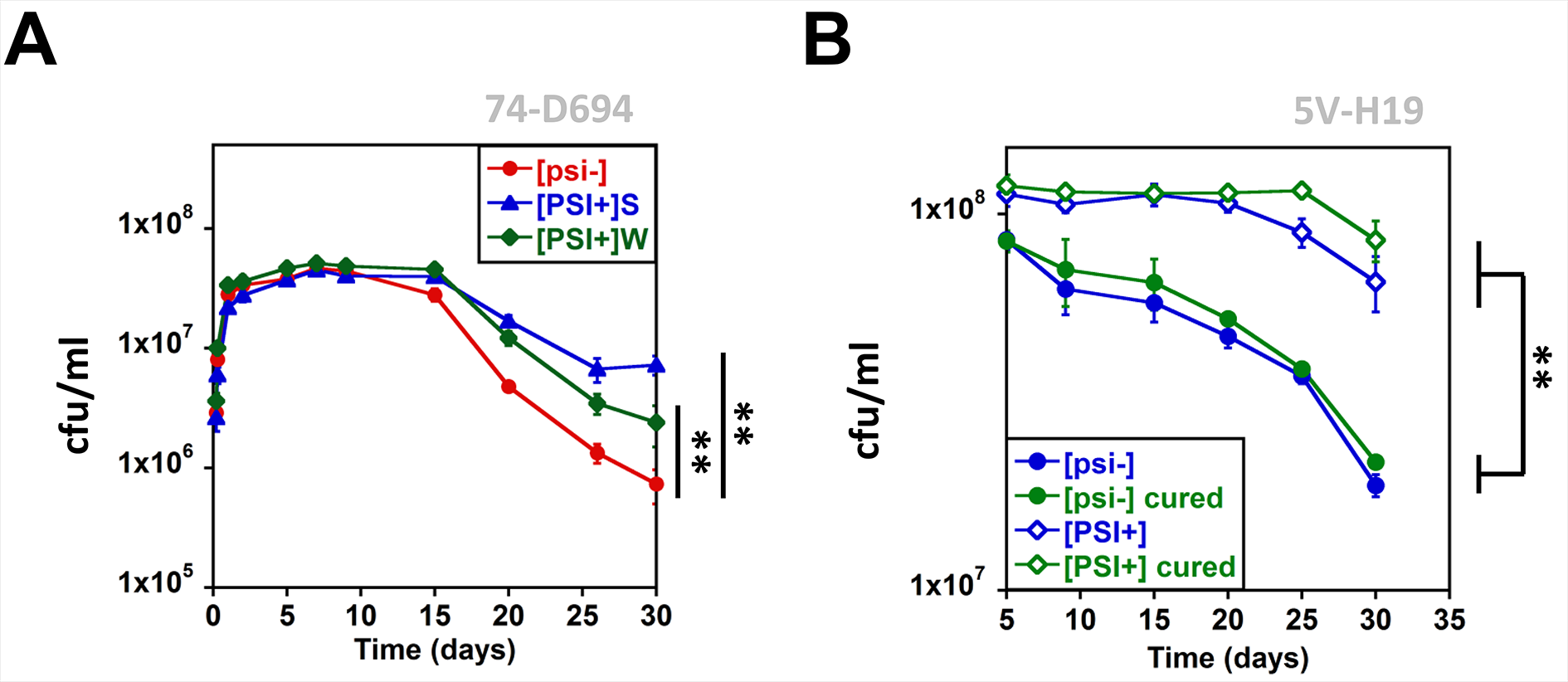

Supplement: S1 Fig — (A) Same as Fig 1B. Data points are the mean cfu numbers ±SE of five independent cultures. Statistical comparison of the growth curves was performed using a permutation test (see Materials and Methods; ** indicate p-values < 0.01). (B) Same as Fig 1F. Data points are the mean cfu numbers ±SE of four to six independent cultures. Statistical comparison of the growth curves was performed using a permutation test (see Materials and Methods; ** indicate p-values < 0.01). (TIF) [file pone.0184905.s001.tif]

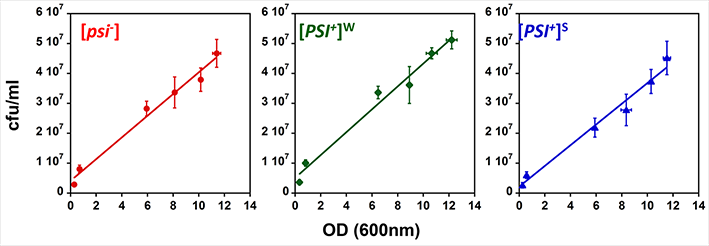

Supplement: S2 Fig — A linear correlation between optical density measurements and cfu numbers (data points are from Fig 1A and 1B) was observed at OD600nm<12. (TIF) [file pone.0184905.s002.tif]

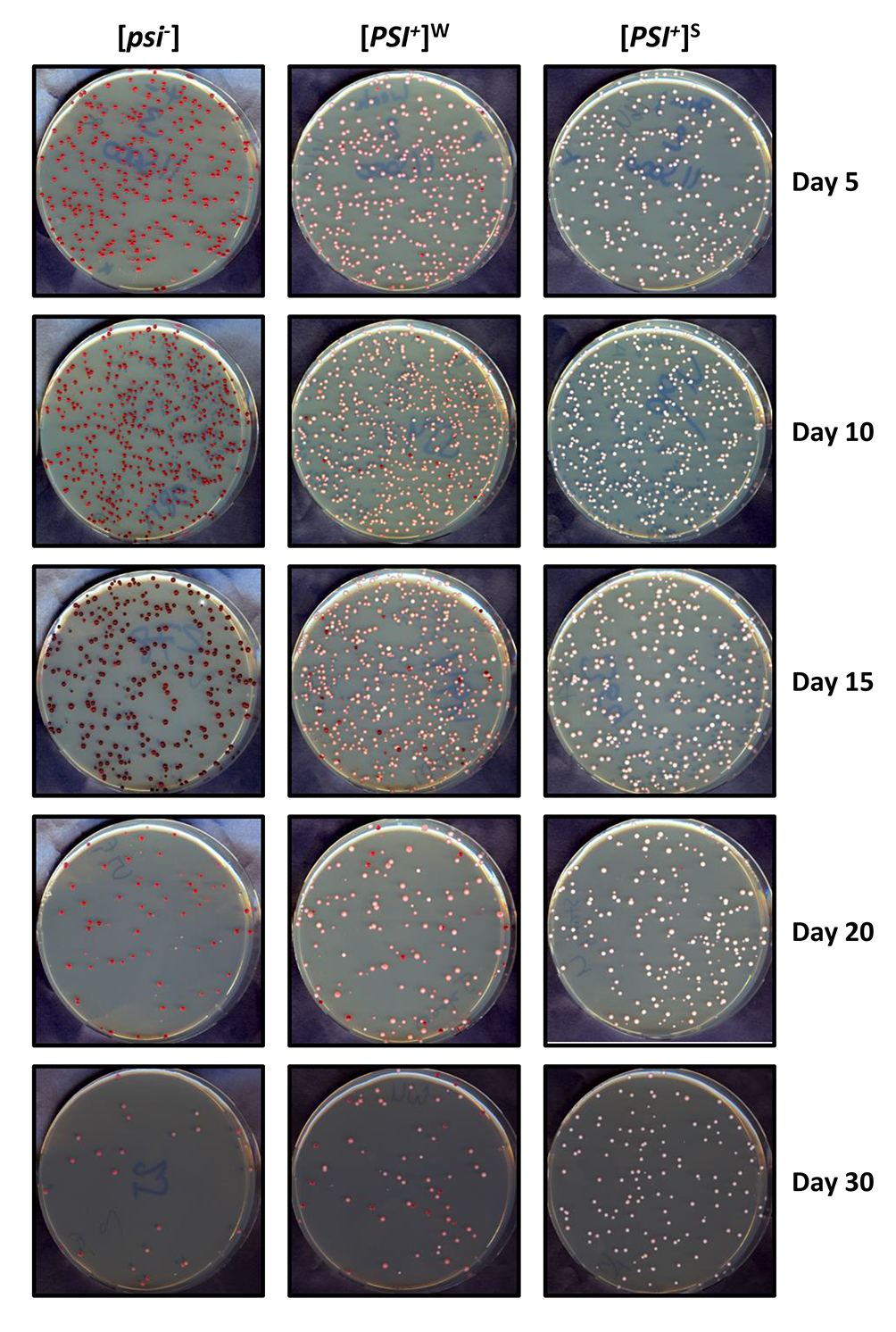

Supplement: S3 Fig — [psi-], [PSI+]S or [PSI+]W 74-D694 cells incubated at 30°C under agitation for up to 30 days were periodically plated on ¼-YPD plates to assess their prion phenotypes. As expected, no spontaneous formation of [PSI+] colonies occurred in the [psi-] cultures. The [PSI+]S variant was stably maintained in all cells while the [PSI+]W variant was lost in less than 5% of the cells. (TIF) [file pone.0184905.s003.tif]

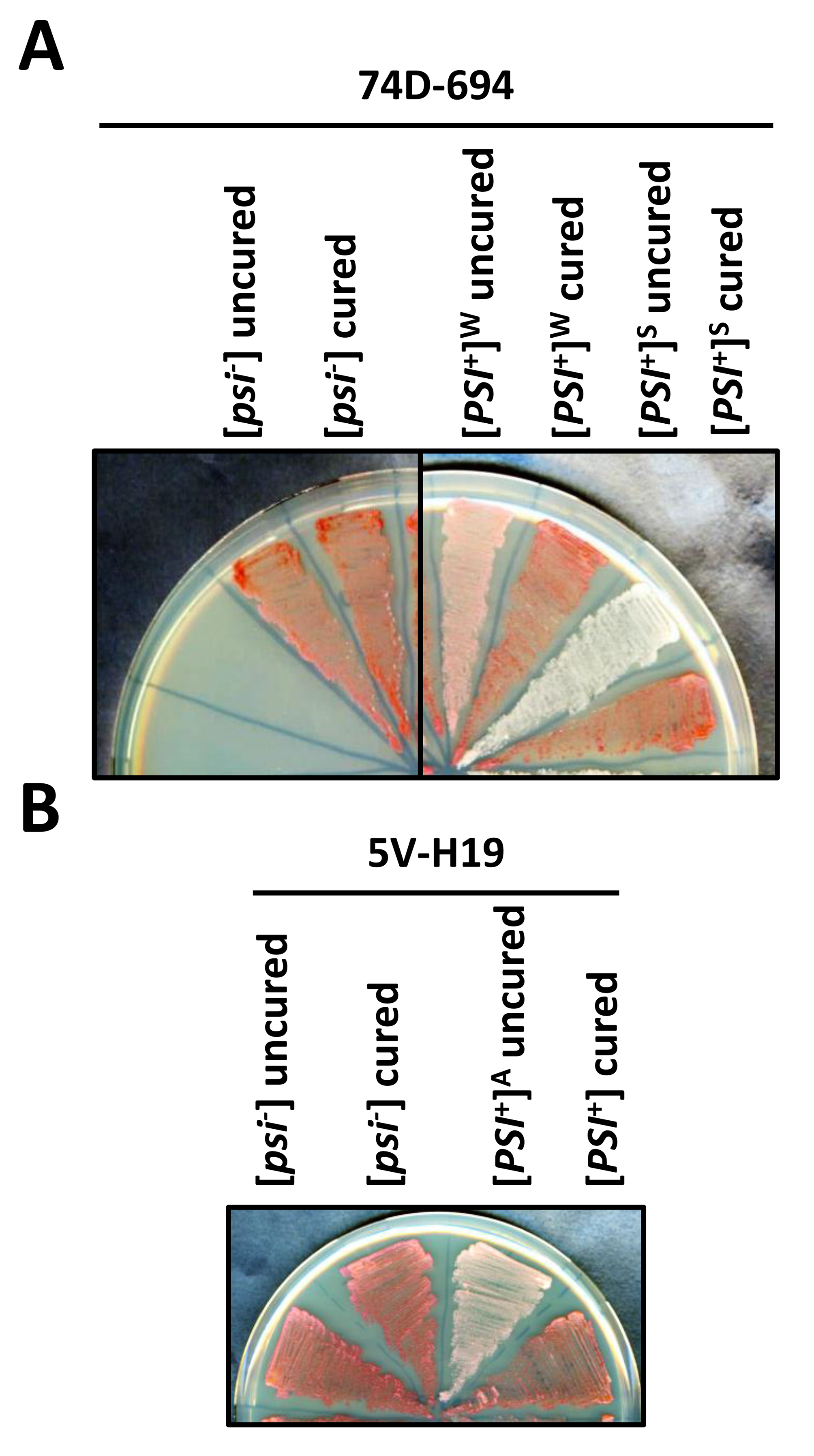

Supplement: S4 Fig — Representative (A) 74-D694 (W, weak [PSI+]; S, strong [PSI+]) or (B) 5V-H19 ([PSI+] clone A; lower panel) [psi-] and [PSI+] clones, either left untreated or treated with guanidine hydrochloride (see Materials and Methods), were streaked on ¼-YPD plates to assess their prion phenotypes. (TIF) [file pone.0184905.s004.tif]

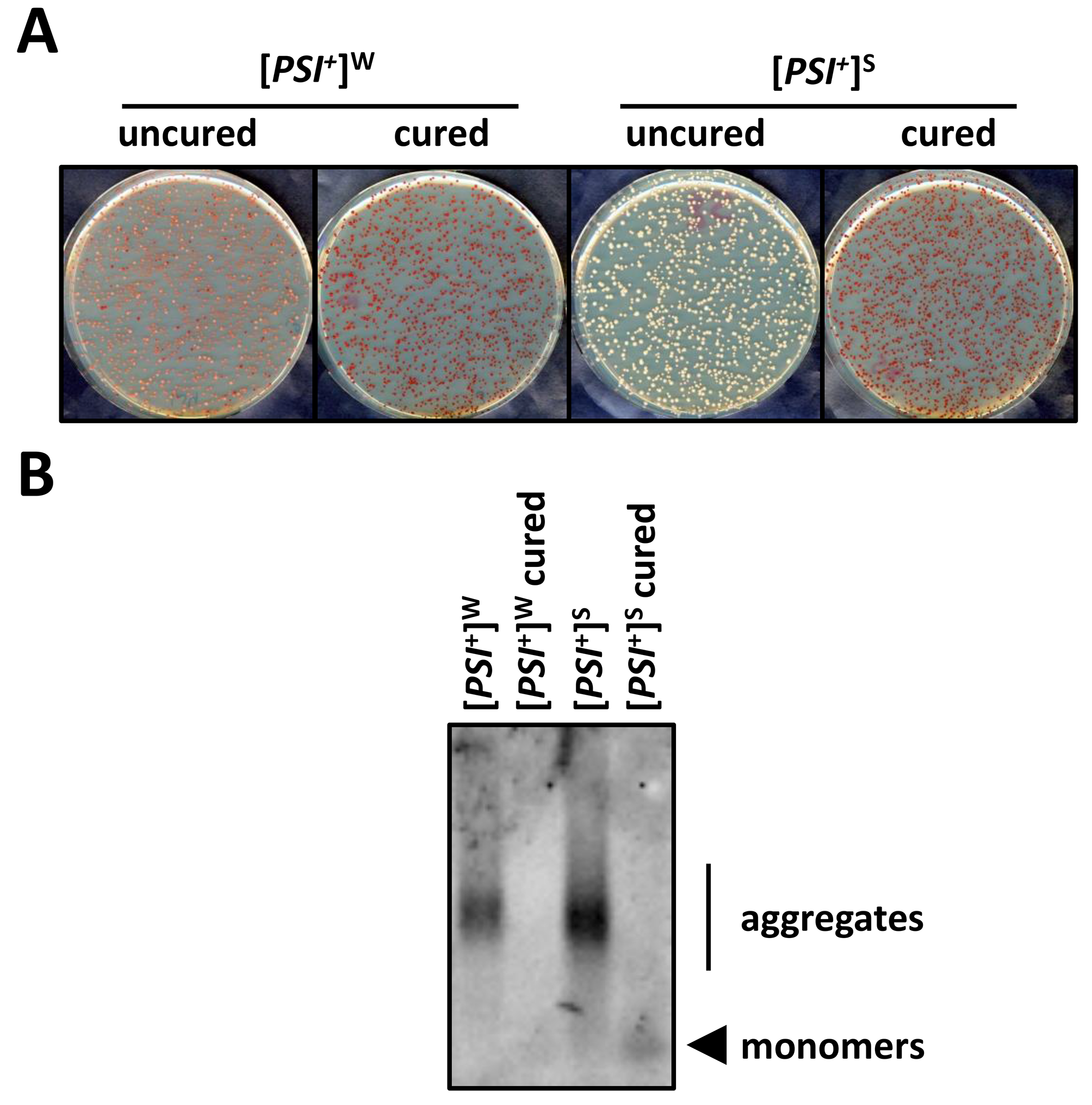

Supplement: S5 Fig — Exponentially growing uncured or guanidine hydrochloride-cured [PSI+]W or [PSI+]S 74-D694 cells were inoculated into fresh YPDA medium and incubated at 30°C under agitation for 30 days. (A) Aliquots of the cultures were plated on ¼-YPD plates to assess their prion phenotypes. (B) Cell lysates prepared from each culture were analyzed by SDD-AGE followed by immunoblotting using anti-Sup35p antibodies. The position of Sup35p monomers (fast migrating species) and aggregates (slow migrating species) is indicated. (TIF) [file pone.0184905.s005.tif]

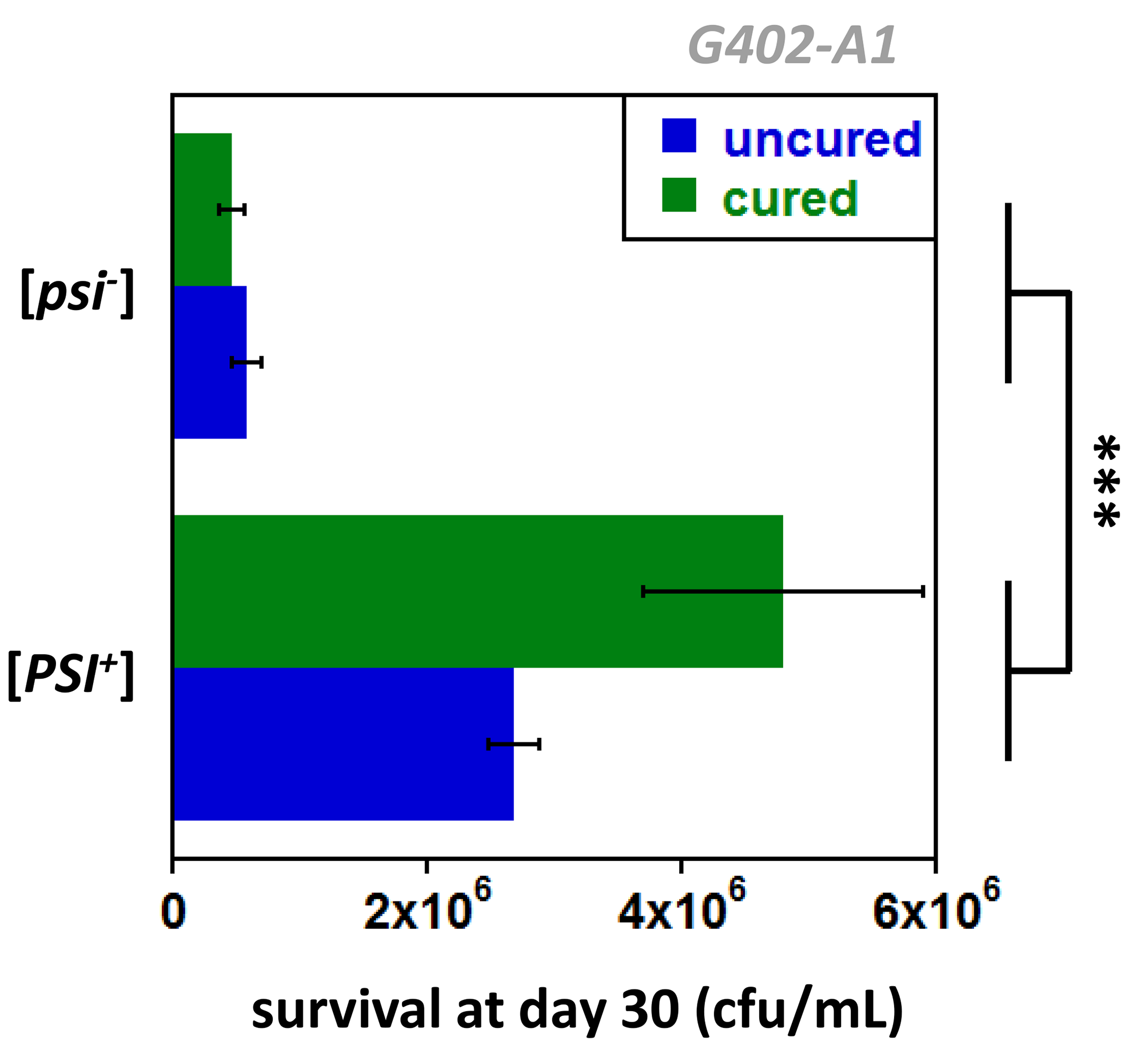

Supplement: S6 Fig — Exponentially growing [psi-] and [PSI+] G402-A1 cells, that were previously cured with guanidine hydrochloride or not, as indicated, were inoculated in fresh YPDA medium and incubated at 30°C under agitation for 30 days. The number of cfu was then determined by serial dilutions and plating. Data represent the mean ±SE of four independent cultures (*** indicate p-values <0.001, unpaired two-tailed Student’s t-test). (TIF) [file pone.0184905.s006.tif]
